# Extracellular vesicles enriched in connexin 43 promote a senescent phenotype in bone and synovial cells contributing to osteoarthritis progression

**DOI:** 10.1038/s41419-022-05089-w

**Published:** 2022-08-05

**Authors:** Marta Varela-Eirín, Paula Carpintero-Fernández, Amanda Guitián-Caamaño, Adrián Varela-Vázquez, Alejandro García-Yuste, Agustín Sánchez-Temprano, Susana B. Bravo-López, José Yañez-Cabanas, Eduardo Fonseca, Raquel Largo, Ali Mobasheri, José Ramón Caeiro, María D. Mayán

**Affiliations:** 1 CellCOM Research Group, Instituto de Investigación Biomédica de A Coruña (INIBIC), Servizo Galego de Saúde (SERGAS), Universidade da Coruña (UDC), A Coruña, Spain; 2 European Research Institute for the Biology of Ageing (ERIBA), University Medical Center Groningen (UMCG), University of Groningen (RUG), Groningen, The Netherlands; 3 Proteomics Laboratory, Instituto de Investigación Sanitaria de Santiago de Compostela (IDIS), Complexo Hospitalario Universitario de Santiago de Compostela (CHUS), Universidade de Santiago de Compostela (USC), Santiago de Compostela, Spain; 4 Department of Orthopaedic Surgery and Traumatology, Complexo Hospitalario Universitario de Santiago de Compostela (CHUS), Universidade de Santiago de Compostela (USC), Santiago de Compostela, Spain; 5 Bone and Joint Research Unit, Rheumatology Department, IIS-Fundación Jiménez Díaz UAM, Madrid, Spain; 6 Research Unit of Medical Imaging, Physics and Technology, Faculty of Medicine, University of Oulu, Oulu, Finland; 7 Department of Regenerative Medicine, State Research Institute Centre for Innovative Medicine, Vilnius, Lithuania; 8 Departments of Rheumatology and Clinical Immunology, University Medical Center Utrecht, Utrecht, The Netherlands; 9 Department of Joint Surgery, First Affiliated Hospital of Sun Yat-sen University, Guangzhou, China; 10 World Health Organization Collaborating Centre for Public Health Aspects of Musculoskeletal Health and Aging, University of Liège, Liège, Belgium

**Keywords:** Osteoarthritis, Predictive markers

## Abstract

The accumulation of senescent cells is a key characteristic of aging, leading to the progression of age-related diseases such as osteoarthritis (OA). Previous data from our laboratory has demonstrated that high levels of the transmembrane protein connexin 43 (Cx43) are associated with a senescent phenotype in chondrocytes from osteoarthritic cartilage. OA has been reclassified as a musculoskeletal disease characterized by the breakdown of the articular cartilage affecting the whole joint, subchondral bone, synovium, ligaments, tendons and muscles. However, the mechanisms that contribute to the spread of pathogenic factors throughout the joint tissues are still unknown. Here, we show for the first time that small extracellular vesicles (sEVs) released by human OA-derived chondrocytes contain high levels of Cx43 and induce a senescent phenotype in targeted chondrocytes, synovial and bone cells contributing to the formation of an inflammatory and degenerative joint environment by the secretion of senescence-associated secretory associated phenotype (SASP) molecules, including IL-1ß and IL-6 and MMPs. The enrichment of Cx43 changes the protein profile and activity of the secreted sEVs. Our results indicate a dual role for sEVs containing Cx43 inducing senescence and activating cellular plasticity in target cells mediated by NF-kß and the extracellular signal-regulated kinase 1/2 (ERK1/2), inducing epithelial-to-mesenchymal transition (EMT) signalling programme and contributing to the loss of the fully differentiated phenotype. Our results demonstrated that Cx43-sEVs released by OA-derived chondrocytes spread senescence, inflammation and reprogramming factors involved in wound healing failure to neighbouring tissues, contributing to the progression of the disease among cartilage, synovium, and bone and probably from one joint to another. These results highlight the importance for future studies to consider sEVs positive for Cx43 as a new biomarker of disease progression and new target to treat OA.

## Introduction

Osteoarthritis (OA) is a complex disease characterized by articular cartilage degradation and degeneration and dysfunction of the entire joint [1, 2]. OA includes low-grade joint inflammation, cellular phenotypic changes and extracellular matrix-degradation [3]. Consistent with other wound-healing disorders, synovium and cartilage from OA patients show an accumulation of senescent cells [4–6] and an overactivity of the gap junction protein connexin 43 (Cx43) [7–10], together with loss of the fully mature phenotype [7, 11–13].

Connexins are key players in cellular signalling and intercellular communication between adjacent (gap junction channels) and nearby cells (hemichannels) [14]. In addition to their channels functions, connexins contain cytoplasmic domains involved in the recruitment of proteins to their C-terminal domains (CTD) that control different signalling pathways, which can occur independently of their channel activity [15]. Further, Cx43 has been found in different cellular compartments including the nucleus [16], and regulates gene expression by interacting with transcription factors (TFs) [17]. Cx43 is also found in tunnelling nanotubes (TNTs) [18, 19] and small extracellular vesicles (sEVs) [20].

Cx43 and cellular senescence are involved in tissue regeneration [7]. However, both have also been reported to impede tissue repair and consequently promote age-related diseases (ARD), including osteoarthritis [21, 22]. Senescence is considered a stress response and accumulation of senescent cells during OA has been involved in cartilage degradation, presumably mediated by the pro-inflammatory factors secreted by these cells, known as the senescence-associated secretory phenotype (SASP) [4, 23, 24], that also activates dedifferentiation and reprogramming of surrounding cells [7, 25, 26]. We have recently reported that Cx43 overactivity in chondrocytes from OA patients (OACs) leads to the accumulation of senescent cells [7, 22].

sEVs (including microvesicles and exosomes) are membrane-based structures, which carry and deliver bioactive molecules [27–29]. sEVs released by chondrocytes were reported to facilitate chondrogenesis and the maintenance of cartilage stability [30]. However, it has been shown that sEVs derived from IL1-ß stimulated synovial fibroblast induce OA-like changes [31] and sEVs secreted from senescent cells from patients can induce senescence and tissue degeneration in healthy chondrocytes and young joints of a surgical model of post-traumatic OA [24].

In this study, we show that Cx43 is upregulated in sEVs released by OACs from patients. In addition, we demonstrate that exosomal Cx43 changes the protein cargo in sEVs, and plays a dual role by activating senescence and dedifferentiation in joint target cells, contributing to the spread and progression of the disease.

## Results

### sEVs secreted by chondrocytes from OA patients are enriched in Cx43

**Fig. 1:**
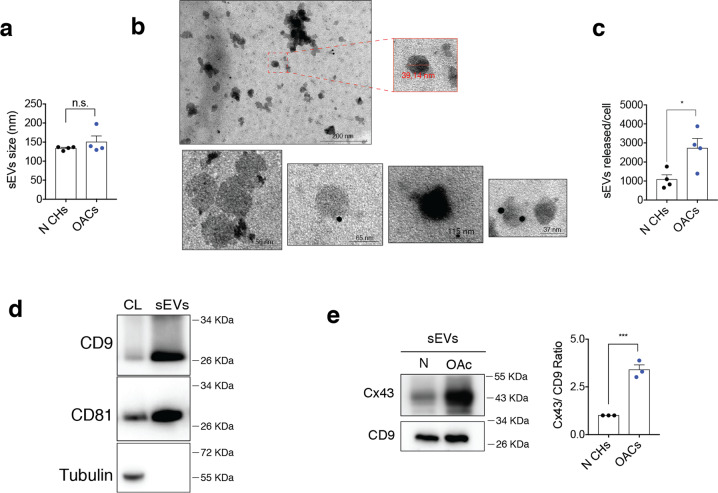
Human osteoarthritic chondrocytes secrete sEVs containing high levels of Cx43. **a** Average size in nm of sEVs released by healthy (N CHs) and OA-derived chondrocytes, determined by nanoparticle tracking analysis (NTA). *n* = 4, Student’s *t* test. **b** Representative electron microscopy images of CD9-immunogold labelling from sEVs derived from OA chondrocytes. **c** Number of sEVs released by healthy and OA chondrocytes after 72 h, detected by NTA. *n* = 4, Student’s *t* test. **d** Representative images of the sEVs markers CD9 and CD81 in sEVs derived from OA chondrocytes. **e** sEVs derived from OA chondrocytes display increased levels of Cx43 when compared to sEVs derived from healthy chondrocytes. *n* = 3, Student’s *t* test. Data are expressed as mean ± S.E.M., *P < 0.05, ***P < 0.0001.

sEVs from patients were isolated by differential ultracentrifugation and characterized by transmission electron microscopy (TEM) and nanoparticle tracking analysis (NTA) (Fig. 1a–c). The data showed that sEVs were 30–200 nm in diameter (Fig. 1a) and cup-shaped morphology typical of exosomes (Fig. 1b). Also, NTA data showed that OACs released significantly more sEVs than chondrocytes from healthy donors (Fig. 1c). Immunoblot analysis showed the enrichment of different exosome-related proteins (CD9, CD81) in our sEVs preparations (Fig. 1d), with absence of proteins related to intracellular compartments such as tubulin (Fig. 1d). Interestingly, Cx43 protein levels were significantly overrepresented (>3 folds) in sEVs released by chondrocytes from patients in comparison with healthy donors (Fig. 1e).

### sEVs enriched in Cx43 increase cellular plasticity by inducing ERK1/2 and EMT signalling

**Fig. 2:**
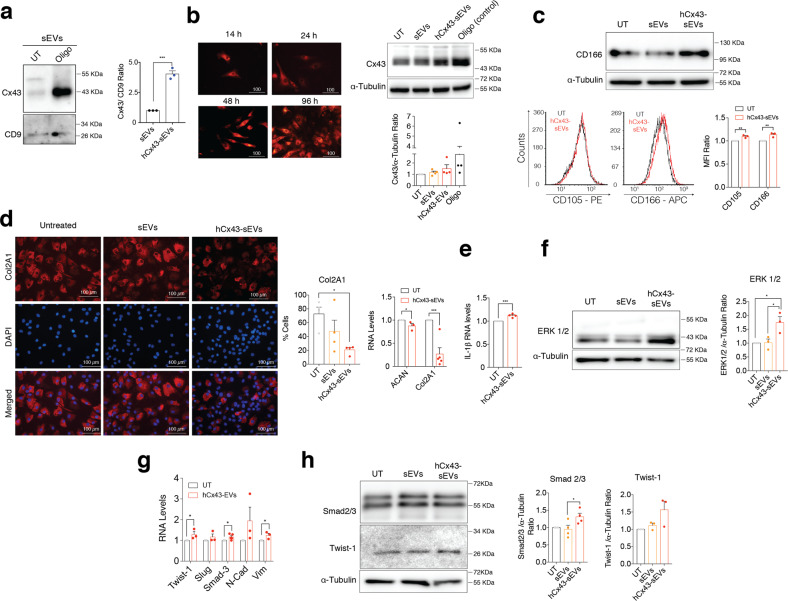
Exosomal Cx43 induces dedifferentiation in target chondrocytes. **a** Cx43 levels detected by WB in sEVs released by OA-derived chondrocytes treated with oligomycin (Oligo). *n* = 3, Student’s *t* test. **b** Cx43-positive sEVs increased Cx43 levels in target chondrocytes treated for 48 h. Oligomycin (Oligo) treatment (5 µM, 48 h) was used as positive control for Cx43 overexpression. *n* = 4. **c** WB and flow cytometry analysis shows increased levels of the mesenchymal markers CD105 and CD166 in OA-derived chondrocytes after 48-h treatment with sEVs derived from Oligo-treated chondrocytes. *n* = 3, Student’s *t* test. **d** Immunofluorescence for collagen type II (Col2A1) showed a significant decreased in OA-derived chondrocytes treated with hCx43-sEVs for 48 h (*n* = 4, one-way ANOVA). On the right, RNA expression of the main cartilage ECM markers ACAN and Col2A1 also showed a significant downregulation after a 48-h treatment with hCx43-sEVs (*n* = 3, Student’s *t* test). **e** IL-1β overexpression in OA-derived chondrocytes treated with sEVs derived from Oligo-treated chondrocytes. *n* = 3, Student’s *t* test. **f** MAPK/ERK pathway is activated in OA-derived chondrocytes after hCx43-sEVs treatment, as detected by higher ERK1/2 levels detected by WB. *n* = 3, one-way ANOVA. **g** Correlated to gene expression, protein levels of the EMT-transcription factors Smad2/3 (*n* = 4) and Twist-1 (*n* = 3) were increased in OA-derived chondrocytes after a 48-h treatment with hCx43-sEVs. Student’s *t* test. **h** The treatment with Cx43-positive sEVs was correlated with overexpression of several EMT-related transcription factors. *n* = 3, Student’s *t* test. Data are expressed as mean ± S.E.M., *P < 0.05, **P < 0.01, ***P < 0.0001.

We exposed human primary healthy chondrocytes to oligomycin (Oligo) to increase total Cx43 protein levels as previously reported [7, 22]. The treatment with oligomycin also increased Cx43 protein levels in the secreted sEVs (hCx43-sEVs) (Fig. 2a). sEVs internalization was tested in primary cultures using Dil-labelled sEVs, which were detected at least until 96 h after treatment (Fig. 2b). Interestingly, the treatment with hCx43-sEVs secreted by Oligo-stimulated chondrocytes lead to increased levels of Cx43 in healthy chondrocytes (Fig. 2b). On the other hand, hCx43-sEVs treatment also induced chondrocyte dedifferentiation detected by increased levels of CD105 and CD166 (Fig. 2c). Changes in cellular phenotype associated with the loss of the fully differentiated phenotype and accumulation of senescent cells after incubation with hCx43-sEVs were confirmed by immunofluorescence and gene expression of Collagen type II (Col2A1) and aggrecan (ACAN) (Fig. 2d). qPCR data showed an increase in the levels of the catabolic factor involved in OA progression IL-1ß (Fig. 2e). Further, enriched hCx43-sEVs secreted by Oligo-stimulated chondrocytes induced an increase in ERK1/2 signalling (Fig. 2f) and subsequent expression of the epithelial-mesenchymal transition (EMT) TFs Twist-1, Slug and Smad3 and the EMT biomarkers N-cadherin and vimentin (Fig. 2g). The increase of EMT-inducing TFs (EMT-TFs) was confirmed by western blot (Fig. 2h) and nuclear translocation of Twist-1 (Suppl. Fig. 1).

**Fig. 3:**
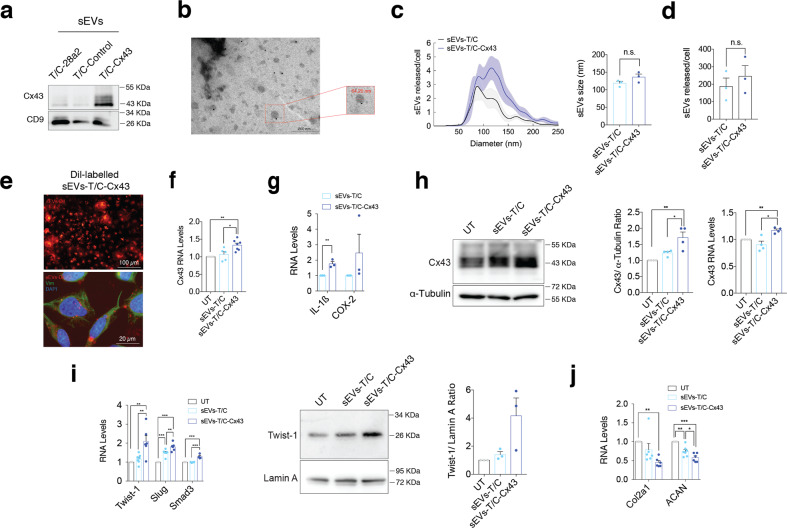
sEVs derived from human osteoarthritic chondrocytes promote OA-phenotype in target chondrocytes. **a** The human chondrocyte cell line T/C-28a2 overexpressing Cx43 released sEVs with higher Cx43 content as detected by WB. **b** Representative electron microscopy image of CD9-immunogold labelling from sEVs released by Cx43-overexpressing T/C-28a2 cells. **c** NTA analysis showing the average size of sEVs (in nm) derived from T/C-28a2 chondrocytes (sEVs-T/C) and Cx43-overexpressing T/C-28a2 chondrocytes (sEVS-T/C-Cx43), collected after 72 h. *n* = 3, Student’s *t* test. **d** NTA analysis of the number of sEVs released by Cx43-overexpressing T/C-28a2 cells and compared to non-transfected T/C-28a2 cells sEVs. *n* = 3, Student’s *t* test. **e** Red Dil-labelled sEVs isolated from Cx43-overexrpessing T/C-28a2 cells were detected in T/C-28a2 chondrocytes after 48-h treatment. Co-staining with vimentin antibody (green) and DAPI (blue) were performed to visualize the target cells. **f** Treatment of T/C-28a2 cells with high-Cx43 sEVs (sEVs-T/C-Cx43) for 48 h promoted Cx43 gene overexpression in target cells (*n* = 5–7, one-way ANOVA), as well as the expression of the inflammatory genes IL-1β and COX-2 (*n* = 3, Student’s *t* test) (**g**). **h** Treatment of T/C-28a2 cells with sEVs derived from Cx43-overexpressing T/C-28a2 chondrocytes (sEVs-T/C-Cx43) for 48 h increased both protein and gene expression levels in target chondrocytes. *n* = 4, one-way ANOVA. **i** When OA-derived chondrocytes were treated with sEVs-T/C-Cx43 for 48 h, EMT-markers gene (*n* = 5–7) and protein (*n* = 3) expression was upregulated, together with decreased gene expression levels of Col2A1 and ACAN (**j**, *n* = 6); one-way ANOVA. Data are expressed as mean ± S.E.M., *P < 0.05, **P < 0.01, ***P < 0.0001.

To mitigate any effects derived from oligomycin treatment, T/C-28a2 chondrocytes were transfected with a vector to overexpress Cx43 to mimic the phenotype of OACs [7]. sEVs released by T/C-28a2 chondrocytes overexpressing Cx43 (sEVs-T/C-Cx43) contained significantly higher levels of Cx43 (Fig. 3a). sEVs were visualized by TEM (Fig. 3b) and showed diameter between 30–200 nm (Fig. 3c). NTA data indicated that T/C-28a2 cells overexpressing Cx43 did not release significantly more sEVs than wild-type T/C-28a2 (Fig. 3d). The effect of hCx43-EVs on inducing dedifferentiation of chondrocytes was confirmed by exposing T/C-28a2 cells to sEVs-T/C-Cx43. Internalization of Dil-labelled T/C-derived sEVs is shown on Fig. 3e. The addition of sEVs-T/C-Cx43 for 48 h increased Cx43 (Fig. 3f) and SASP-related factors gene expression, such as IL-1ß and COX-2 (Fig. 3g). Further, sEVs-T/C-Cx43 also increased Cx43 levels (Fig. 3h) and EMT-TFs in primary OACs (Fig. 3i), decreasing the synthesis of Col2A1 and ACAN (Fig. 3j).

### Exosomal Cx43 induces cellular senescence turning chondrocyte into pro-inflammatory cells

**Fig. 4:**
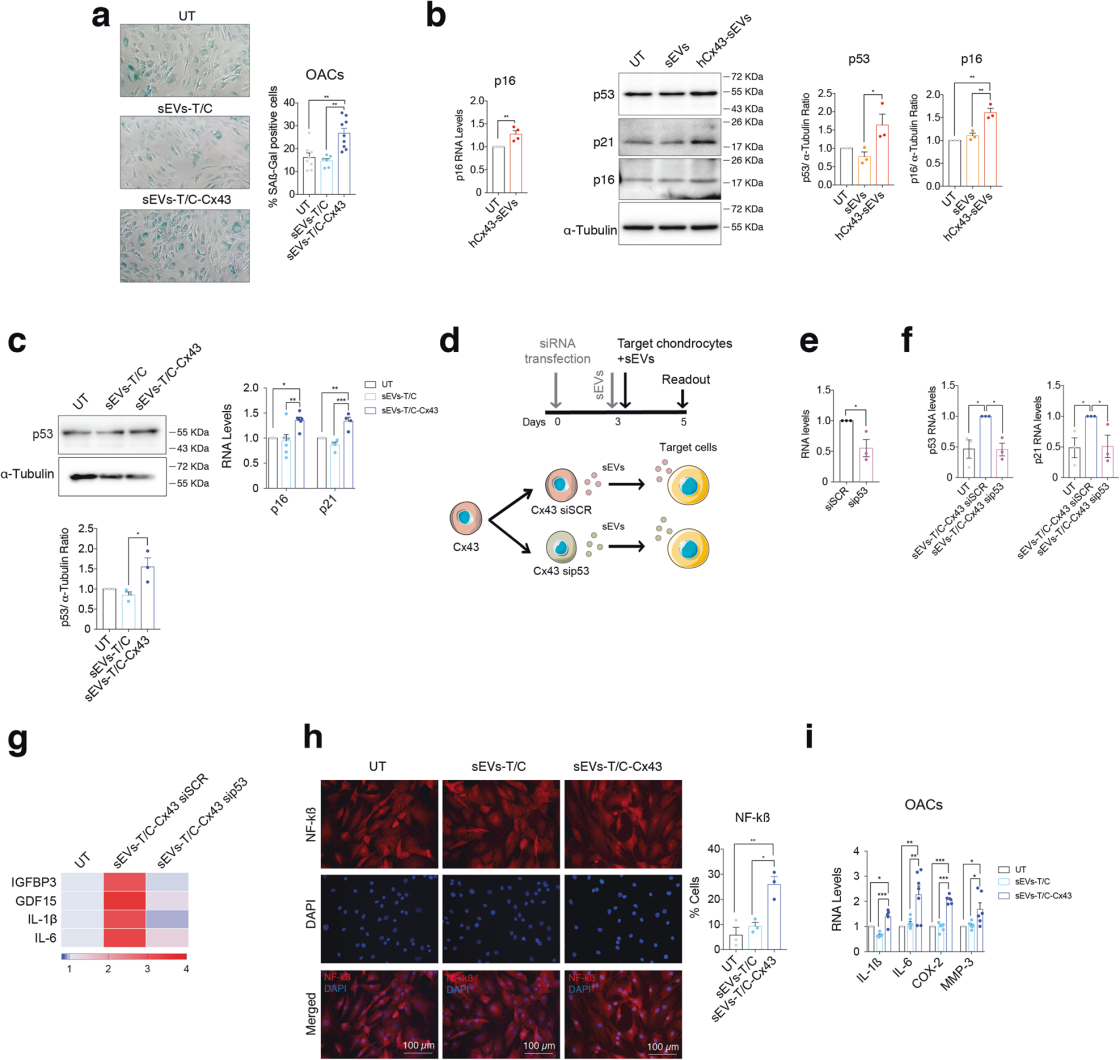
Exosomal Cx43 promotes a senescence inflammatory phenotype in target chondrocytes. **a** Treatment of OA-derived chondrocytes with sEVs derived from Cx43-overexpressing T/C-28a2 chondrocytes (sEVs-T/C-Cx43) for 48 h increased the number of senescence-associated β-galactosidase (SA-β-Gal) positive cells. *n* = 6–9, one-way ANOVA. **b** The gene expression levels of the senescence marker p16 gene (*n* = 4, Student’s *t* test), as well as the protein levels as p53 (*n* = 3, one-way ANOVA), p21 (*n* = 2) and p16 (*n* = 3, one-way ANOVA) markers were upregulated in OA-derived chondrocytes after a 48-h treatment with sEVs derived from Oligo-treated OA-derived chondrocytes (hCx43-sEVs). **c** The treatment of OA-derived chondrocytes with sEVs derived from Cx43-overexpressing T/C-28a2 chondrocytes (sEVs-T/C-Cx43) for 48 h lead to increased p53 protein levels (*n* = 3, one-way ANOVA) and the gene expression of p16 (*n* = 5, one-way ANOVA) and p21 (*n* = 4, one-way ANOVA). **d** Schematic representation and timeline followed for siRNA transfection in Cx43-overexpressing T/C-28a2 chondrocytes. siRNA targeting p53 (sip53) and a siRNA control (siSCR) were transfected into the Cx43-overexpressing T/C-28a2 cell line. sEVs were collected 72 h after transfection and immediately used to treat primary chondrocytes for 48 h. **e** P53 RNA-analysis in sip53 knockdown in T/C28a2 chondrocytes overexpressing Cx43, compared to siSCR in the same cell type. *n* = 3, Student’s *t* test. **f** RNA expression levels of p53, p21 in OA primary chondrocytes after treatment with sEVs for 48 h. **g** Heatmap showing the expression levels of different p53-targets and NF-kB SASP factors after sEVs treatment for 48 h, *n* = 3. **h** sEVs derived from Cx43-overexpressing T/C-28a2 chondrocytes (sEVs-T/C-Cx43) promoted NF-kβ activation in OA-derived chondrocytes, as detected by the increased number of nuclear NF-kβ positive cells. *n* = 3, one-way ANOVA. **i** RNA expression analysis of different SASP components showed upregulation in OA-derived chondrocytes after 48-h treatment with sEVs-T/C-Cx43. *n* = 4–7, one-way ANOVA. Data are expressed as mean ± S.E.M., *P < 0.05, **P < 0.01, ***P < 0.0001.

The exposure of sEVs-T/C-Cx43 for 48 h increased ß-galactosidase activity in OACs (Fig. 4a). The senescent factor p53, its target gene p21, and the senescence biomarker p16^INK4a^ were upregulated when OACs were incubated with hCx43-sEVs secreted by Oligo-stimulated chondrocytes (Fig. 4b) or with sEVs-T/C-Cx43 (Fig. 4c). We tested whether the induction of senescence and the pro-inflammatory phenotype by Cx43-enriched sEVs could be dependent on the p53 pathway (Fig. 4d). T/C-28a2 chondrocytes stably overexpressing Cx43 were transfected with sip53 (Fig. 4e), followed by sEVs isolation and treatment of OACs. As shown in Fig. 4f, OA chondrocytes treated with sEVs isolated from sip53 cells failed to induce both p53 and p21 expression. In addition, p53-related SASP factors (GFBP3, GDF15), as well as, NF-kB-dependent SASP factors (IL-6, IL-1ß) followed the same trend when p53 was knocked-down (Fig. 4g). These results indicate that the effect of exosomal Cx43 on target cells would be at least partially dependent on p53 activity. Activation of NF-kß and the transcription of pro-inflammatory cytokines in the presence of sEVs-T/C-Cx43 was tested (Fig. 4h, i) showing NF-kß nuclear translocation together with an increase of SASP factors.

### Cx43 regulates the protein cargo in chondrocyte-derived sEVs

**Fig. 5:**
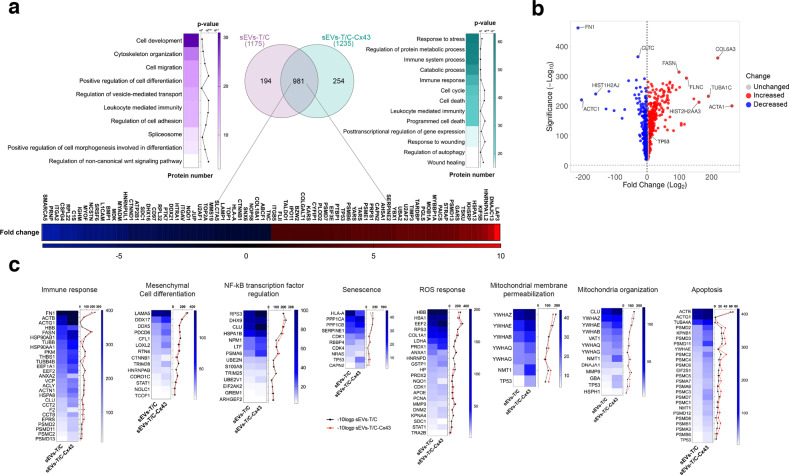
Proteomic analysis of proteins isolated from sEVs derived from T/C-28a2 chondrocytes with (sEVs-T/C-Cx43) or without (sEVs-T/C) Cx43. **a** sEVs were isolated by ultracentrifugation and the protein content was analysed by mass spectrometry (TripleTOF 6600 System). Venn diagram showing the number of proteins quantified in both groups (sEVs-T/C in the left, sEVs-T/C-Cx43 in the right), and the heatmap of upregulated and downregulated proteins in the bottom. **b** Volcano plot (VolcaNoseR) illustrating the spectral count fold change and its significance in the common proteins shared by sEVs-T/C and sEVs-T/C-Cx43. Overrepresented proteins in sEVs-T/C-Cx43 are displayed as red dots, while blue dots represent proteins that are increased in the sEVs-T/C group. **c** Heatmaps representing the spectral count values and the −10log *p*-value of proteins involved in NF-kß transcription factor regulation, mitochondrial membrane permeabilization and organization, ROS response, immune response, apoptosis, mesenchymal cell differentiation and senescence. Black lines correspond to −10log *p*-value of sEVs-T/C and red lines to −10log *p*-value sEVs-T/C-Cx43.

**Fig. 6:**
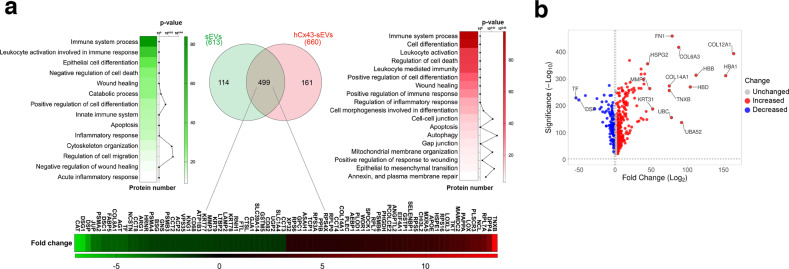
Proteomic analysis of proteins isolated from sEVs derived from untreated human osteoarthritic chondrocytes (sEVs) or Cx43-enriched after oligomycin treatment (hCx43-sEVs). **a** Venn diagram indicating the number of proteins identified in the proteomic analysis, and the heatmap of enriched pathways present in each group (*n* = 4). 114 proteins were exclusively found in sEVs isolated from OA-derived chondrocytes (left) and 161 were only identified in hCx43-sEVs (right). 499 common proteins (middle circle) were identified in both samples. Heatmaps represent significant function and pathways enriched in exclusive and common detected proteins, and analysed using STRING enrichment analysis (Gene Ontology and KEGG) based on quantitatively upregulated and downregulated proteins. **b** Volcano plot (VolcaNoseR) illustrating the correlation of fold change derived from the semiquantitative parameter spectral count and its significance, of the common proteins presents in both groups (sEVs from OA-derived chondrocytes-derived and sEVs derived from hCx43).

To determine whether Cx43 affects the protein cargo of sEVs derived from human chondrocytes, we isolated sEVs with low and high levels of Cx43 and examined the protein content by mass spectrometry (Figs. 5 and 6). The top molecular networks detected in control sEVs and Cx43-sEVs and predicted by Gene Ontology analysis (GO) are shown in Fig. 5a. The analysis identified 981 common proteins that were differentially expressed between sEVs with and without Cx43 enrichment (Fig. 5a and Tables S2, S3), 254 proteins were exclusively present in Cx43-sEVs (Tables S2, S4) and 194 proteins exclusively present in control sEVs (Tables S2, S5). Comparison of the total proteins found in sEVs derived by healthy chondrocytes (T/C-28a2; sEVs-T/C) and Cx43-sEVs (sEVs-T/C-Cx43) revealed an enrichment in proteins related with stress response and immune system, metabolic and catabolic processes or cell cycle, among others, when Cx43 is present (Fig. 5a). Specifically, p53 is enriched more than 3 folds in sEVs-T/C-Cx43 (Fig. 5b, Tables S2, S3, S6). On the other hand, Cx43-sEVs contain lower levels of MVP (−1.31 folds), which has been involved in downregulation of SRC activity signalling. Interestingly, we also identified several proteins exclusively present in sEVs-T/C-Cx43 involved in protein transport and in the unfolded protein response (UPR), as DNAJB9 (ERdj4) or HYOU1 (GRP170), a process activated in ER stressed cells to ensure cell survival and involved in senescence (Table S2). Senescent cells display ER stress due to increase protein synthesis, as well as upregulation of UPR pathways as an attempt to counteract the proteostasis imbalance [32]. In addition, chronic ER stress has been associated with OA [33, 34]. We also observed the presence of ubiquitin-specific protease USP10 (Tables S2, S3, S6), involved in DNA-damage response, maintenance of genomic stability and NF-kB activation [35]. Importantly, the mammalian target of rapamycin (mTOR) protein, as well as its activator LAMTOR1, were only detected in sEVs-T/C-Cx43 (Table S2). Remarkably, mTOR, which acts as a positive SASP regulator [36–38], is overexpressed in OA cartilage, and mTOR ablation was correlated with increased autophagy and protection from cartilage degeneration in OA animal models [39]. Matrix metalloproteinase 1 (MMP-1) was exclusively detected in sEVs derived from Cx43-overexpressing chondrocytes (hCx43-sEVs) (Table S2) and is both associated with senescence as one of the main SASP components [40] and OA progression [41]. MMP-2 was also overrepresented in sEVs-T/C-Cx43 by 1.3 folds (Tables S2, S3, S6). Critical proteins involved in wound-healing pathway including Cx43, mTOR, MYH10, LOX or MAPK3 and proteins classified within the immune response pathway were significantly overrepresented in sEVs-T/C-Cx43 (Tables S2, S2, S6), We have also detected the chemokine stromal cell-derived factor 1 (CXCL12), which has been showed to be overexpressed in senescence prostate fibroblasts [42] (Table S2).

We detected several proteins that interact with Cx43 in sEVs enriched in Cx43 such as cytoskeletal proteins (Table S2) (Fig. 5b). HSPA8 (Heat Shock Cognate Protein 70), which is 1.5 fold overrepresented in sEVs-T/C-Cx43 (Tables S2, S3, S6), was described to interact with Cx43 and modulate cyclin D1 nuclear translocation, affecting cell cycle progression [43, 44]. In addition, previous results from our group have detected HSPA8 as a Cx43-interacting protein in human primary articular chondrocytes, with overrepresentation in moderate OA compared to healthy or early OA stages [45].

Overrepresented common proteins between sEVs-T/C and sEVs-T/C-Cx43 are displayed in Fig. 5b and Table S6 (see Tables S2 and S3), and some differential pathways are represented in Fig. 5c. In summary, overrepresented proteins in sEVs-T/C-Cx43 are included in processes such as immune response, epithelial/mesenchymal differentiation, senescence and inflammatory response (Fig. 5c). The disease/functional analysis component of differentially expressed in sEVs with and without Cx43 showed differences in pathways related with immune response, apoptosis, senescence, ROS response, EMT or mitochondrial activity, among others (Fig. 5c and Table S6).

Similar results were obtained in sEVs enriched in Cx43 derived from OACs (Fig. 6). The proteomic analysis identified 499 proteins that were commonly shared but differentially expressed due to the Cx43-enrichment (Fig. 6a and Tables S7 and S8), with 161 proteins exclusively present in sEVs enriched in Cx43 as a result of the oligomycin treatment (Tables S7, S9) and 114 proteins exclusively present in control sEVs (Fig. 6a and Tables S7, S10). GO analysis showed a Cx43-related enrichment in proteins related with the immune system, wound healing, autophagy, inflammation, mitochondrial membrane organization and EMT (Fig. 6a). Interestingly, we were able to detect enrichment in gap junction-related proteins, including several α/β-Tubulin proteins, GNAI2 and GNAS (Tables S7, 8, S11). Regarding catabolic processes, we detected some proteins exclusively present in the Cx43-enriched sEVs such as PRSS3P2 (Trypsin-2) and ARSB (Arysulfatase B), which is involved in the degradation of chondroitin-4-sulfate. Similarly to sEVs derived from T/C-28a2 cells overexpressing Cx43, MMP-2 was also overrepresented in Cx43-enriched sEVs isolated from OACs by 1.4 folds (Tables S7, 8, S11). We have also detected enrichment of mesenchymal markers in sEVs derived from OACs when Cx43 was upregulated, including VIM, VCL or FN1 (Tables S7, 8, S11). UPR and stress-related pathways were highly overrepresented within the proteins detected in Cx43-sEVs, including several members of the heat shock protein family (e.g. HSP90B1) (Fig. 6b, Tables S7, S8, S11). Persistent DNA-damage response (DDR) signalling is extensively described in senescence and associated with cell cycle arrest, mainly through chronic activation of p53. Interestingly, several proteins associated with DDR were detected only in Cx43-enriched sEVs, including XRCC5, XRCC6, SFRP1 or PARP1 (Tables S8, S9, S11). In addition, when we compared the proteins detected in Cx43-enriched and control sEVs groups, we detected an overrepresentation of proteins from the p53-signalling pathway including SERPINE1 (PAI-1), THBS1, CYCS and IGFBP3 (Tables S7, S8, S11). Remarkably PAI-1, which is overrepresented in Cx43-enriched sEVs, is associated with aging and was described to be a senescent marker and a mediator of senescence in ARD [46].

### Cx43-sEVs derived from osteoarthritic chondrocytes induce cellular senescence in bone and synovial cells

**Fig. 7:**
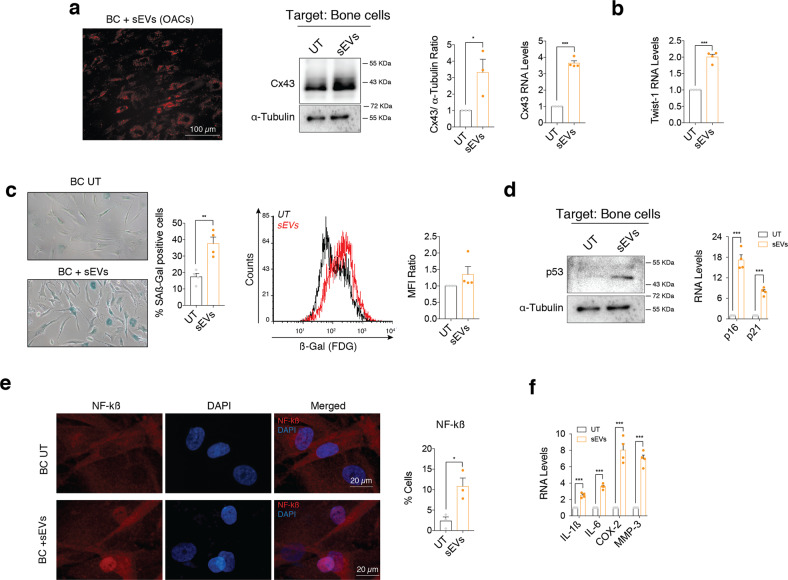
sEVs secreted by OA-derived chondrocytes promote inflammation and senescence in bone cells. **a** Red Dil-labelled sEVs isolated from OA-derived chondrocytes were detected in bone cells (BC) after 48-h treatment. Cx43 protein (middle; *n* = 3, Student’s *t* test) and RNA (right, *n* = 4; Student’s *t* test) levels were upregulated in bone cells treated with sEVs derived from OA-derived chondrocytes, as determined by western blot and RT-qPCR, respectively. **b** RNA levels of the EMT-transcription factor Twist-1 were also significantly upregulated in bone cells after a 48-h treatment with sEVs from OA chondrocytes. *n* = 4, Student’s *t* test. **c** Senescence-associated β-galactosidase activity (SA-β-Gal) was detected by microscopy (left; *n* = 4, Student’s *t* test) and flow cytometry (right; *n* = 4 Student’s *t* test) in bone cells treated for 48 h with sEVs derived from OA-derived chondrocytes. **d** Western blot analysis showed increase p53 protein levels in bone cells treated with sEVs isolated from OA-derived chondrocytes. On the right, gene expression levels of the senescence markers p16 and p21 were upregulated in BC after a 48-h treatment with sEVs from OA chondrocytes. *n* = 4, Student’s *t* test. **e** Treatment of BC with sEVs isolated from OA-derived chondrocytes for 48 h promoted NF-kβ nuclear translocation, as detected by immunofluorescence. *n* = 3, Student’s *t* test. **f** Overexpression of SASP factors IL-1β, IL-6, COX-2 and MMP-3 in BC treated for 48 h with sEVs isolated from OA-derived chondrocytes. *n* = 4, Student’s *t* test. Data are expressed as mean ± S.E.M., *P < 0.05, **P < 0.01, ***P < 0.0001.

As OA affects the whole joint, we decided to investigate the role of sEVs secreted by OACs on bone and synovial cells. Bone cells (BC) were isolated from subchondral bone from donors and were exposed to sEVs derived from OACs (Figs. 1e and 7), revealing an increase in Cx43 levels in target BC (Fig. 7a), which correlated with increased levels of Twist-1 gene expression (Fig. 7b). In addition, we detected increased levels ß-galactosidase activity in BC (Fig. 7c), which was accompanied by increased levels of p53 protein and overexpression of p21 and p16 genes (Fig. 7d). NF-kß activation was confirmed by immunofluorescence (Fig. 7e) and by SASP overexpression (Fig. 7f).

**Fig. 8:**
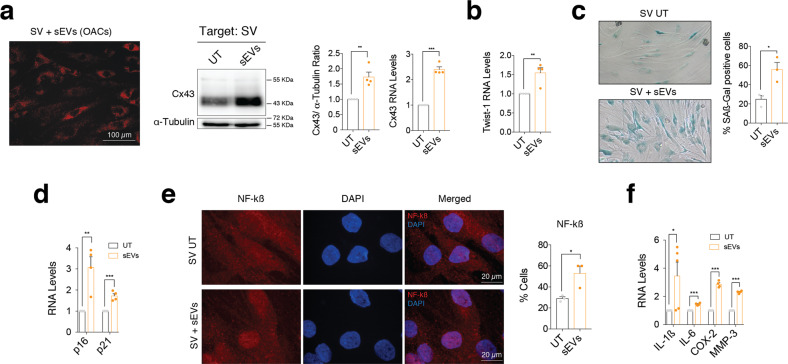
sEVs derived by OA-derived chondrocytes promote inflammation and senescence in synovial cells. **a** Red Dil-labelled sEVs isolated from OA-derived chondrocytes were detected in synovial cells (SV) after 48-h treatment. Cx43 protein and RNA levels were upregulated in synovial cells (SV) treated with sEVs derived from OA-derived chondrocytes, as detected by western blot and RT-qPCR, respectively. *n* = 4; Student’s *t* test. **b** RNA levels of the EMT-transcription factor Twist-1 were significantly upregulated in synovial cells after a 48-h treatment with sEVs isolated from OA-derived chondrocytes. *n* = 4, Student’s *t* test. **c** Senescence-associated β-galactosidase activity (SA-β-Gal) levels were upregulated synovial cells treated for 48 h with sEVs derived from OA-derived chondrocytes. *n* = 3, Student’s *t* test. **d** The gene expression levels of the senescence markers p16 and p21 were upregulated in SV after a 48-h treatment with sEVs derived from OA-derived chondrocytes. *n* = 4, Student’s *t* test. **e** Treatment of synovial cells (SV) with sEVs isolated from OA-derived chondrocytes promoted NF-kβ nuclear translocation, as detected by immunofluorescence. *n* = 3, Student’s *t* test. **f** Overexpression of SASP factors IL-1β, IL-6, COX-2 and MMP-3 in SV treated for 48 h with sEVs isolated from OA-derived chondrocytes. *n* = 4–5, Student’s *t* test. Data are expressed as mean ± S.E.M., *P < 0.05, **P < 0.01, ***P < 0.0001.

To further investigate the effects of osteoarthritic sEVs in joint degeneration, OACs-sEVs enriched in Cx43 were added to synovial cells (SV) isolated from the synovium of OA donors (Fig. 8a). We detected overexpression of Cx43 levels (Fig. 8a), increased gene expression of Twist-1 (Fig. 8b) and increased levels of ß-galactosidase activity in targeted SV (Fig. 8c). Twist-1 activation was also detected in SV after treatment with sEVs-T/C-Cx43 (Suppl. Fig. 2). p21 and p16^INK4a^ gene expression were significantly upregulated in SV treated with sEVs derived from OACs (Fig. 8d). OACs-derived Cx43-sEvs promoted NF-kß nuclear translocation (Fig. 8e) and significantly increased the expression levels of SASP genes IL1-ß, IL-6, COX-2 and MMP-3 (Fig. 8f).

**Fig. 9:**
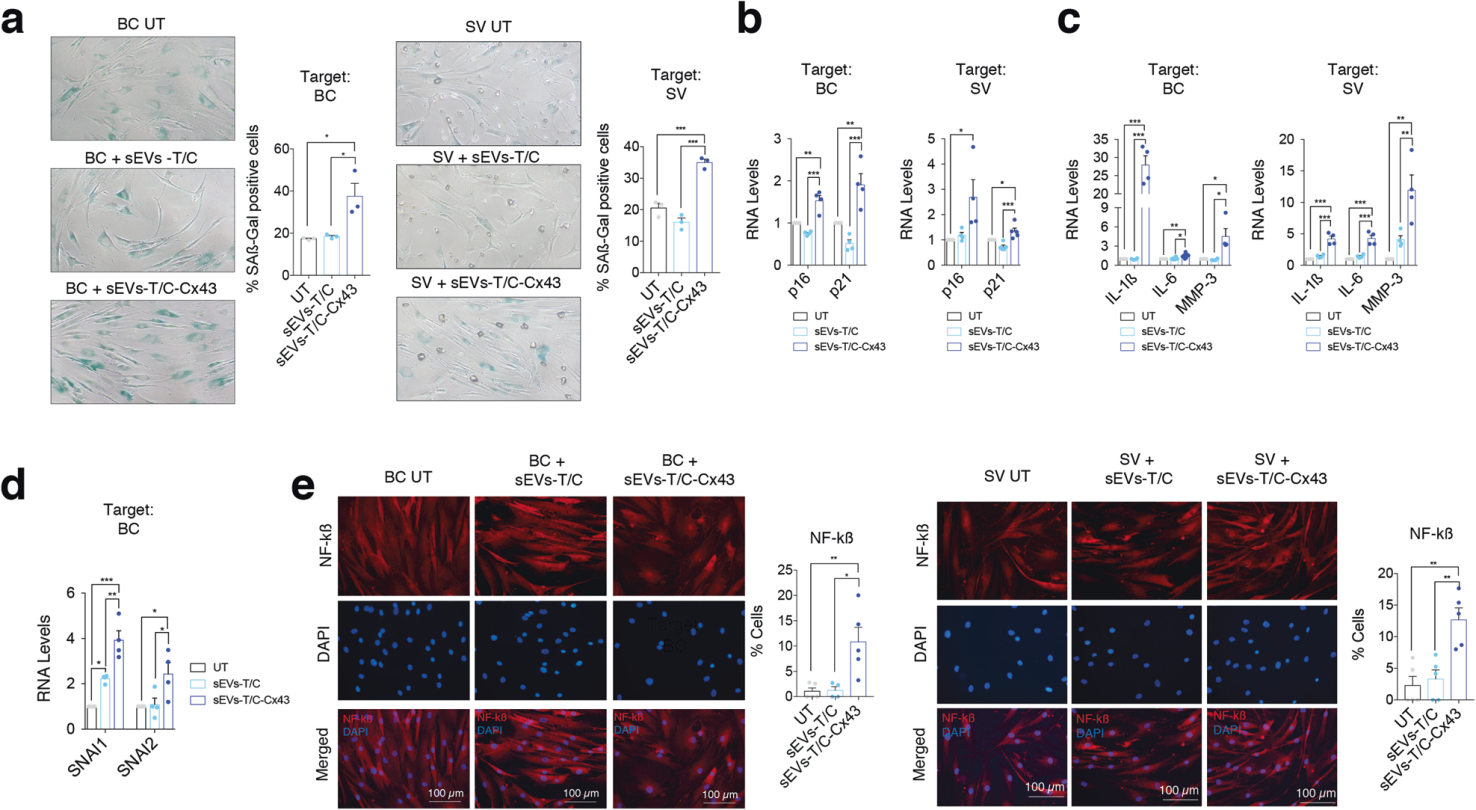
sEVs derived from Cx43-overexpressing human chondrocytes promote OA-phenotype in bone and synovial cells. **a** Senescence-associated β-galactosidase activity (SA-β-Gal) levels were upregulated in both BC (left) and SV (right) treated for 48 h with sEVs derived from Cx43-overexpressing T/C-28a2 chondrocytes. *n* = 3, one-way ANOVA. **b** Treatment of BC (left, *n* = 4) and SV (right, *n* = 4–5) with sEVs derived from Cx43-overexpressing T/C-28a2 chondrocytes (sEVs-T/C-Cx43) for 48 h increased p16 and p21 gene expression levels. One-way ANOVA. **c** IL-1β, IL-6 and MMP-3 expression was upregulated in both bone cells (left) and synovial f cells (right) after treatment with sEVs derived from Cx43-overexpressing T/C-28a2 chondrocytes (sEVs-T/C-Cx43) for 48 h. *n* = 4, one-way ANOVA. **d** Treatment of BC (*n* = 4) with sEVs derived from Cx43-overexpressing T/C-28a2 chondrocytes (sEVs-T/C-Cx43) for 48 h increased SNAI1 and SNAI1 gene expression levels. One-way ANOVA. **e** The treatment of BC (left) and SV (right) with sEVs isolated from Cx43-overexpressing T/C-28a2 chondrocytes (sEVs-T/C-Cx43) promoted NF-kβ nuclear translocation. *n* = 4–5, one-way ANOVA. Data are expressed as mean ± S.E.M., *P < 0.05, **P < 0.01, ***P < 0.0001.

To further confirm that exosomal Cx43 induces senescence in BC and SV, we treated these cells with sEVs-T/C-Cx43, resulting in increased ß-galactosidase activity (Fig. 9a) and upregulation of p16 and p21 (Fig. 9b). Furthermore, sEVs-T/C-Cx43 treatment was accompanied by an overexpression of different SASP factors in comparison with cells treated with sEVs with low levels of Cx43 (Fig. 9c) and induced the expression of EMT-TFs in target cells (Fig. 9d and Suppl. Fig. 2). As shown in Fig. 9e, BC and SV treated with sEVs-T/C-Cx43 increased NF-kß activity. Altogether, these data indicate that Cx43 in sEVs enhances cellular senescence and increases cell plasticity in targeted chondrocytes, bone and synovial cells.

## Discussion

In this study, we demonstrate that sEVs isolated from OA patients are enriched in Cx43, and exosomal Cx43 participates in disease progression by promoting a pro-inflammatory and degenerative condition with the joint environment.

It has been reported that sEVs can modulate the phenotype of recipient cells [47–50]. In fact, sEVs secreted by senescent chondrocytes can transfer features of senescence to non-senescent chondrocytes and suppress cartilage tissue formation [24]. However, the mechanism by which sEVs enhance senescence in normal chondrocytes has not been elucidated yet. Our data indicate that sEVs isolated from OACs are highly enriched in Cx43, which triggers senescence in target cells by increasing the expression of p53, p21 and p16 (Figs. 1e, 4b, c, 7d, 8d, 9b). Interestingly, p53 knockdown in Cx43-overexpressing chondrocytes partially inhibited the sEVs’ senescence-triggering effect in recipient OACs, also affecting both NF-kß and p53-related SASP (Fig. 4d–i) [51].

Many non-canonical connexin pathways occurs through a complex interactome where the CTD plays critical roles [52]. Importantly, besides enhancing senescence, the hCx43-sEVs also drives dedifferentiation by activation of ERK and EMT-TFs (Figs. 2f–h, 3i, 7b, 8b, 9d). However, we cannot discard that the promotion of cell dedifferentiation was triggered by SASP (from a secondary senescence mechanism) [53–55]. On the other hand, dedifferentiated cells express higher levels of ECM remodelling and pro-inflammatory factors which also may drive senescence, opening feedback loop amplification events involved in tissue degradation in patients [7]. Cellular senescence, dedifferentiation and reprogramming are damage responses aimed to orchestrate tissue repair. Transient senescence was shown to be essential for tissue repair, while chronic activation comprises regeneration and disrupts normal tissue structure and function [56]. We have already demonstrated that chronic activation of Cx43 activates dedifferentiation and senescence in human chondrocytes [7].

Our results showed that Cx43 is able to change the protein component of sEVs. Based on the proteomic analysis, we propose that Cx43 affects the quantity and protein composition of sEVs released by chondrocytes (see Supplementary Tables S2–S9) promoting the activation of different signalling pathways related to stress response, senescence, UPR, immune response inflammation or catabolism in target cells (Figs. 5 and 6), contributing to tissue degradation by modulating cellular plasticity and promoting phenotypic switch of chondrocytes, synovial and bone cells (Figs. 2–7). The proteomic analysis revealed proteins involved in senescence, including proteins related to the UPR or SASP-related proteins such as mTOR, which plays an essential role in promoting the secretory phenotype in senescent cells [36]. We also identified UPR and other stimuli that are well characterized as senescence inducers, including mitochondrial dysfunction, oxidative stress and certain cytokines [57]. It is tempting to speculate that Cx43 may interact and promote the enrichment of sEVs in proteins involved in the enhancement or exacerbation of senescence and regulation of the inflammatory SASP via NF-kß signal transduction pathways. Our analysis also identified proteins involved in wound healing, such as DCN (Decorin) in Cx43-enriched sEVs derived from chondrocytes, which is binds to collagen fibrils and it is involved in wound healing. Our data clearly demonstrates that Cx43 changes sEVs activity and affect their protein cargo. However, further studies will be useful to discern if Cx43 directly regulates the sEVs protein cargo by the interaction of its CTD with different proteins (signalling hub function) or if the regulation occurs in and indirect manner by modulating gene expression in the target cells. Nevertheless, our data is in line with previous studies where it was demonstrated that exosomes isolated from OA synovial fluid stimulated the release of inflammatory cytokines, chemokines and metalloproteinases in target cells [58].

A recent study indicates the crucial role of exosomal Cx43 in chemotherapy resistance and migration of glioma cells suggesting that Cx43 may hold promise as a therapeutic target for glioblastoma [59]. Particularly, the authors found that temozolomide glioma resistant-cells secrete higher levels of Cx43 in sEVs promoting an increase in proliferation and Bcl-2 expression but reducing Bax and cleaved-caspase 3 in target cells. On the other hand, Cx43 secretion trough sEVs derived from cardiomyocytes participates in ischaemic heart disease [60]. A different study demonstrated that the presence of Cx43 in sEVs reduces the cardiotoxicity of the chemotherapeutic drug doxorubicin by decreasing doxo-induced oxidative stress in the heart [61]. These data indicate that the role of exosomal Cx43 appears to be dependent upon cellular context and probably has disease-dependent roles.

Recently, several strategies have been implemented to eliminate senescent cells in ARD such as the use of senolytics (e.g. navitoclax or UBX0101) [21]. Also, peptide drugs that target Cx43 are being used to improve wound healing and for the treatment of ARD [62–65]. In OA, the downregulation of Cx43 in human osteoarthritic chondrocytes restores chondrocyte redifferentiation and decreases the propensity of chondrocytes to undergo cellular senescence by downregulating p53/p21 and inhibiting the nuclear translocation of NF-κB [7, 22]. Furthermore, the downregulation of Cx43 in other inflammatory and wound healing disorders halts disease progression and restores tissue regeneration [62, 63, 66]. Different studies indicate that Cx43 may represent a new therapeutic target against inflammation during the progression of fibrosis and different degenerative disorders [67]. In fact, the use of targeted therapy such as specific peptides of the CTD of Cx43, may be useful for the management and treatment of ARD such as macular generation (Peptide5, ACT1), hepatic and cardiac injury (P5), corneal injury (ACT1), gingival wound healing (Gap19) or spatial short-term memory (Gap19) [68]. These new therapeutic approaches are impacting in various diseases with special focus on small peptides to specifically target Cx43-binding domains such as Src-interacting regions at the Cx43 C-terminal domain, that exerts neuroprotective effects in vivo by inhibiting hemichannel activity and tissue damage likely mediated by c-Src in astrocytes [69]. Therefore, the use of compounds or small molecules that downregulate Cx43 may be attractive candidates to the development of new therapeutic approaches to effectively decrease inflammation, cellular senescence and restore tissue regeneration in ARD.

OA is a heterogeneous disease with several molecular and clinical phenotypes [70, 71]. After tissue damage, the transient activation of senescence limits the replication of old and damaged cells and boost the secretion of SASP factors, that contribute to enhance tissue regeneration and repair [72] and stemness [54]. On the other hand, chronic activation of Cx43 or senescence leads to the accumulation of senescent cells driving ARD [73]. Our results demonstrated that Cx43 is a key player on sEVs derived by senescent chondrocytes and probably participates in disease progression. Our results demonstrated that Cx43-sEVs efficiently spread cellular senescence in different target cell types including chondrocytes, bone and synovial cells, and may be involved in the spread of OA from one joint to another. These hCx43-sEVs and their content may also have prognostic or diagnostic value for OA or serve as an indicator to predict the therapeutic response to particular therapies.

## Methods

### Cell culture

Cartilage collection and processing were performed as previously [7, 22]. The study was conducted with the approval of the institutional ethics committee (C.0003333, 2012/094 and 2015/029) and after informed consent were obtained. The cartilage of healthy donors (*n* = 6) was obtained from individuals who suffered a knee or hip fracture with no history of joint disease. The isolation and culture of primary chondrocytes from human knees and femoral heads from adult donors undergoing joint surgery (*n* = 12) was performed as previously described [7, 22]. Briefly, cartilage was rinsed with saline, separated from the subchondral bone, diced and incubated in 0.5 mg/mL trypsin-EDTA solution (Life Technologies) for 10 min at 37 °C, with shaking. After the trypsin solution was removed, samples were incubated in a 2 mg/mL collagenase type IV solution (Sigma-Aldrich) for 18 h at 37 °C, with shaking. The chondrocytes suspension was filtered through a 100 µm nylon cell strainer and cultured in DMEM supplemented with 100 U/mL penicillin (Life Technologies), 100 µg/mL streptomycin (Life Technologies) and 10% Foetal Bovine Serum (Life Technologies Gibco). Synoviocytes were isolated from the synovial tissue of human donors (*n* = 4). Briefly, synovial tissue was chopped into small pieces and cultured at 5% CO_2_ and 37 °C. Primary cells attached to the 100-mm dish and were cultured in RPMI 1640 with 10% FBS, 100 U/mL penicillin, 100 µg/mL streptomycin and 1% insulin-transferrin-selenium. Bone cells were isolated by explant culture and cultured in DMEM supplemented with 20% inactivated FBS, 100 U/mL penicillin and 100 µg/mL streptomycin (*n* = 5). The T/C-28a2 chondrocyte cell line, kindly donated by Dr. Goldring (the Hospital for Special Surgery, New York, USA), was cultured in DMEM supplemented with 10% FBS, 100 U/ml penicillin and 100 μg/ml streptomycin. The T/C-28a2 cell line was transfected with a plasmid to overexpress Cx43 (kindly provided by Arantxa Tabernero from the Institute of Neuroscience of Castilla y León, University of Salamanca, Salamanca, Spain) as previously described [7].

### RNA silencing

A commercial small interfering RNA (siRNA) against p53 (Santa Cruz Biotechnology) and a control siRNA (Santa Cruz Biotechnology) were transfected into the Cx43-overexpressing T/C-28a2 chondrocytes with the siRNA Transfection Reagent (Santa Cruz Biotechnology) following the manufacturer’s instructions. sEVs were collected 72 h after transfection and immediately used for treatments (*n* = 3).

### sEVs isolation and characterization

To isolate small extracellular vesicles (sEVs), including exosomes, the protocol of differential ultracentrifugation [74, 75] was used, with modifications. Cells were plated into two 175 cm^2^ flasks per condition and cultivated until acquire 80–90% confluent, washed with sterile PBS and cultured for 48–72 h in DMEM with no FBS supplementation. Supernatants were collected and centrifuged at 2000 × *g* for 10 min. Pellets were discarded and supernatants were filtered through a 0.22 µm filter, and then ultra-centrifuged at 100,000 × *g* for 90 min at 4 °C in a 70 Ti rotor (Beckman). Supernatants were discarded and sEVs-containing pellets were washed with PBS and centrifuged at 100,000 × *g* for 90 min at 4 °C, and then resuspended in lysis buffer for western blot or PBS for NTA analysis. Pellet-containing sEVs were directly resuspended in culture medium for functional assays.

### Gene expression

Total RNA was isolated using TRIzol reagent according to manufacturer’s instructions (Invitrogen). Next, 200 µl of chloroform were added to all samples, which were then vigorously agitated for 15 s and then incubated for 3 min at room temperature. The RNA was treated with DNase (RNase-free DNase, Invitrogen) to ensure the degradation of DNA in the samples. A total of 1 µg of total RNA per reaction was used to synthesize cDNA with the SuperScript® VILO™ cDNA Synthesis Kit as instructed by the manufacturer (Invitrogen). qRT-PCR was performed with the LightCycler 480 SYBR Green I Master from Roche on a real-time PCR machine (LightCycler® 480 System, Roche). Primer sequences are listed in Table S1. A minimum of *n* = 3 experiment were performed.

### Immunofluorescence

Cell immunofluorescence was performed as previously described [7]. Fixed and membrane-permeabilized cells were incubated with primary antibodies for 1 h at RT, and with fluorescent-secondary antibodies for another 1 h at RT, in dark. Nuclei were stained with 4’,6-diamidino-2- phenylindole dihydrochloride (DAPI; Sigma-Aldrich) for 4 min at RT in the dark. The following primary antibodies were used: Cx43 (Sigma-Aldrich, C6129; 1:500), collagen II (Invitrogen, Thermo Fischer Scientific, MA5-12789; 1:200), NF-κB (Santa Cruz Biotechnology, sc-8008; 1:100) and Twist-1 (Santa Cruz Biotechnology, sc-81417; 1:100). Goat anti-rabbit FITC-conjugated (F-2765; 1:100) and goat anti-mouse Alexa 594-conjugated (A-11032; 1:200) secondary antibodies were used (both from Invitrogen, Thermo Fisher Scientific). Negative controls without primary antibody were performed to test the specificity of each antibody. The samples were analysed on an Olympus BX61 microscope using a DP71 digital camera (Olympus). A minimum of *n* = 3 experiment were performed.

### Western blot

Cells pellets were lysed in ice-cold lysis buffer (150 mM NaCl, 50 mM Tris-HCl pH 7.5, 5 mM EDTA pH 8, 0.5% v/v Nonidet P-40, 0.1% (w/v) SDS, 0.5% (v/v) Sarkosyl) supplemented with 5 µg/ml protease inhibitors cocktail and 1 mM PMSF. Total protein content was determined by Bradford protein assay, and equal amounts of protein were separated in 10% SDS-PAGE and transferred onto a polyvinylidene fluoride (PVDF) membrane (Millipore Co., Bedford, MA). Transference was confirmed staining the membrane with ATX Ponceau S red staining solution (Sigma-Aldrich), followed by blocking with 5% milk in TBS (Tris-Buffered-Saline; 20 mM Tris, 150 mM NaCl) and 0.05% Tween-20 (Sigma-Aldrich). Primary antibody incubation was performed O/N at 4 °C with rotation, and HRP-secondary probing at RT for 1 h. Pierce^TM^ ECL Western Blotting Substrate in an Amersham Imager 600 (GE Healthcare) was used. The following primary antibodies were used: α-tubulin (Sigma-Aldrich, T9026; 1:10,000), Cx43 (Sigma-Aldrich, C6219; 1:1000), Twist-1 (Santa Cruz Biotechnology, sc-81417; 1:100), p16^INK4a^ (Abcam, ab108349; 1:100), p53 (Santa Cruz Biotechnology, sc-126; 1:100), p21 (Santa Cruz Biotechnology, sc-6246; 1:100), ERK (Santa Cruz; 1:200) NF-κB (Santa Cruz Biotechnology, sc-8008; 1:100) and lamin A (Santa Cruz Biotechnology, sc-20680; 1:1000). A minimum of *n* = 3 experiment were performed.

### Senescence-associated β-galactosidase activity

SA-βGal activity was assessed by flow cytometry with the fluorogenic β-galactosidase substrate di-β-D-galactopyranoside (FDG; Invitrogen, Thermo Fisher Scientific) or with the Senescence Cells Histochemical Staining Kit (Sigma-Aldrich), as previously described [7]. A minimum of *n* = 4 experiment were performed for SA-βGal activity assessment.

### Flow cytometry analysis

Cells were fixed with 1% paraformaldehyde and incubated with phycoerythrin (PE)-conjugated anti-human CD105 (Immunostep, 105PE-100T; 1:50) or allophycocyanin (APC)-conjugated anti-human CD166 (Immunostep, 1399990314; 1:50) for 30 min at 4 °C. Events were collected on a FACScalibur^TM^ flow cytometer (Becton Dickinson) with the CellQuest^TM^ Pro software. Forward scatter (FSC) and side scatter (SSC) were used to gate alive cells and discriminate cell debris. Data were analysed with FCS Express 6 Flow software (De Novo Software); *n* = 3 was used for flow cytometry analysis.

### Proteomics and mass spectrometry analysis

sEVs from T/C-282 cells (*n* = 3) or primary chondrocytes from OA donors (*n* = 4) were collected in lysis buffer (150 mM NaCl, 50 mM Tris-HCl (pH 7.5), 5 mM EDTA (pH 8), 0.5% (v/v) Nonidet P-40, 0.1% (w/v) SDS, 0.5% (v/v) N-Lauroylsarcosine) for protein isolation. 100 µg from all conditions were simultaneously concentrated by 10% SDS-PAGE and visualized by Sypro-Ruby fluorescent staining (Lonza). Gel slice were excised and subjected to in-gel manual tryptic digestion following the protocol defined by Shevchenko [76] with minor modifications. Peptides were extracted by performing three 20-min incubation in 40 μL of 60% ACN (Sigma-Aldrich) dissolved in 0.5% formic acid (HCOOH) (Sigma-Aldrich). For mass spectrometry analysis, peptides were analysed by LC-MS/MS using a micro-liquid chromatography system (Eksigent Technologies nanoLC 400, SCIEX) coupled to high-speed Triple TOF 6600 mass spectrometer (SCIEX) with a micro flow source. Peptides were loaded onto a reverse phase column (Chrom XP C18 150 × 0.30 mm, 3 mm particle size and 120 Å pore size; Eksigent, SCIEX), separated and MS data acquired using Analyst TF 1.7.1 software (SCIEX).

### Mass spectrometry data processing and analysis

For identification, data were processed using ProteinPilot^TM^ 5.01 software (SCIEX), which uses the algorithm Paragon^TM^ for database search and Progroup^TM^ for data grouping. Data were searched using a Human specific Uniprot database. False discovery rate (FDR) was performed using a non-lineal fitting method displaying only those results that reported a 1% Global false discovery rate (FDR) or better (shilov IV and Tang WH). The protein Quantification was performed using a Spectral count method. Protein Pilot was searched with a fragment ion mass tolerance of 0.100 Da and a parent ion tolerance of 0.050 Da. MS/MS were normalized between samples using Scaffold 5.0 by the sum of the unweighted spectral counts for each sample method in order to determine a sample-specific scaling factor and then this was applied to all proteins in all the samples. Therefore Scaffold (version Scaffold_5.0.0, Proteome Software Inc., Portland, OR) was used to validate MS/MS-based peptide and protein identifications. Peptide identifications were accepted if they could be established at >95.0% probability by the Percolator posterior error probability calculation [77]. Protein identifications were accepted if they could be established at >99.0% probability and contained at least 1 identified peptides. Protein probabilities were assigned by the Protein Prophet algorithm [78]. Proteins that contained similar peptides and could not be differentiated based on MS/MS analysis alone were grouped to satisfy the principles of parsimony. Proteins sharing significant peptide evidence were grouped into clusters. All calculations were performed using GraphPad Prism 7.0. Enrichment analyses were performed by STRING software version 11.0b (Gene Ontology (GO), KEGG Orthology and Reactome data base). Volcano plot was performed using VolcaNoseR webtool [79] to confirm the presence of two differential groups of proteins.

### Statistical analysis

Statistical analysis was performed with GraphPad Prism software (version 7.0a). Data are represented as mean ± S.E.M. Differences between two groups were estimated with two-tailed unpaired Student’s *t* test, while when more than two groups were compared one-way ANOVA with Tuckey correction was used to estimate the differences among means. A minimum sample size of 3 independent experiments was used in all experiments for statistical comparisons. *P* value <0.05 was considered statistically significant. *P < 0.05, **P < 0.01, ***P < 0.0001.

## Supplementary information

Data Availability Statement

Reproducibility Checklist

Legends

Supplementary Legends

Figure S1

Figure S2

Figure S3

Table S1

Table S2

Table S3

Table S4

Table S5

Table S6

Table S7

Table S8

Table S9

Table S10

Table S11

## Data Availability

The data that support the findings of this study are available from the corresponding authors upon reasonable request.
