# Supplementary material for: Extracellular vesicles enriched in connexin 43 promote a senescent phenotype in bone and synovial cells contributing to osteoarthritis progression
Source: Cell Death Dis. 2022 Aug 5;13(8):681. doi: 10.1038/s41419-022-05089-w (PMC9355945; doi:10.1038/s41419-022-05089-w)
Supplement: Supplementary file 1 — Data Availability Statement [file 41419_2022_5089_MOESM1_ESM.pdf]

Data Availability Statement

The data that support the findings of this study are available from the corresponding authors upon reasonable request.
