# Supplementary material for: Extracellular vesicles enriched in connexin 43 promote a senescent phenotype in bone and synovial cells contributing to osteoarthritis progression
Source: Cell Death Dis. 2022 Aug 5;13(8):681. doi: 10.1038/s41419-022-05089-w (PMC9355945; doi:10.1038/s41419-022-05089-w)
Supplement: Supplementary file 3 — Legends [file 41419_2022_5089_MOESM3_ESM.docx]

**Legends**

**Figure 1**. **Human osteoarthritic chondrocytes secrete sEVs containing high levels of Cx43**. **a**. Average size in nm of sEVs released by healthy (N CHs) and OA-derived chondrocytes, determined by nanoparticle tracking analysis (NTA). n=4, Student’s *t* test. **b**. Representative electron microscopy images of CD9-immunogold labelling from sEVs derived from OA chondrocytes. **c**. Number of sEVs released by healthy and OA chondrocytes after 72 h, detected by NTA. n=4, Student’s *t* test. **d**. Representative images of the sEVs markers CD9 and CD81 in sEVs derived from OA chondrocytes. **e**. sEVs derived from OA chondrocytes display increased levels of Cx43 when compared to sEVs derived from healthy chondrocytes. n=3, Student’s *t* test. Data is expressed as mean ± S.E.M., **P*<0.05, ****P*<0.0001.

**Figure 2. Exosomal Cx43 induces dedifferentiation in target chondrocytes. a**. Cx43 levels detected by WB in sEVs released by OA-derived chondrocytes treated with oligomycin (Oligo). n=3, Student’s *t* test. **b**. Cx43-positive sEVs increased Cx43 levels in target chondrocytes treated for 48 h. Oligomycin (Oligo) treatment (5 µM, 48 h) was used as positive control for Cx43 overexpression. n=4. **c**. WB and flow cytometry analysis shows increased levels of the mesenchymal markers CD105 and CD166 in OA-derived chondrocytes after 48-h treatment with sEVs derived from Oligo-treated chondrocytes. n=3, Student’s *t* test. **d**. Immunofluorescence for collagen type II (Col2A1) showed a significant decreased in OA-derived chondrocytes treated with hCx43-sEVs for 48 h (n=4, one-way ANOVA). On the right, RNA expression of the main cartilage ECM markers ACAN and Col2A1 also showed a significant downregulation after a 48 h-treatment with hCx43-sEVs (n=3, Student’s *t* test). **e**. IL-1β overexpression in OA-derived chondrocytes treated with sEVs derived from Oligo-treated chondrocytes. n=3, Student’s *t* test. **f**. MAPK/ERK pathway is activated in OA-derived chondrocytes after hCx43-sEVs treatment, as detected by higher ERK1/2 levels detected by WB. n=3, one-way ANOVA. **g**. Correlated to gene expression, protein levels of the EMT-transcription factors Smad2/3 (n=4) and Twist-1 (n=3) were increased in OA-derived chondrocytes after a 48-h treatment with hCx43-sEVs. Student’s *t* test. **h**. The treatment with Cx43-positive sEVs was correlated with overexpression of several EMT-related transcription factors. n=3, Student’s *t* test. Data is expressed as mean ± S.E.M., **P*<0.05, ***P*<0.01, ****P*<0.0001.

**Figure 3. sEVs derived from human osteoarthritic chondrocytes promote OA-phenotype in target chondrocytes. a**. The human chondrocyte cell line T/C-28a2 overexpressing Cx43 released sEVs with higher Cx43 content as detected by WB. **b**. Representative electron microscopy image of CD9-immunogold labelling from sEVs released by Cx43-overexpressing T/C-28a2 cells. **c**. NTA analysis showing the average size of sEVs (in nm) derived from T/C-28a2 chondrocytes (sEVs-T/C) and Cx43-overexpressing T/C-28a2 chondrocytes (sEVS-T/C-Cx43), collected after 72 h. n=3, Student’s *t* test. **d**. NTA analysis of the number of sEVs released by Cx43-overexpressing T/C-28a2 cells and compared to non-transfected T/C-28a2 cells sEVs. n=3, Student’s *t* test. **e**. Red Dil-labelled sEVs isolated from Cx43-overexrpessing T/C-28a2 cells were detected in T/C-28a2 chondrocytes after 48-h treatment. Co-staining with vimentin antibody (green) and DAPI (blue) were performed to visualize the target cells. **f**. Treatment of T/C-28a2 cells with high-Cx43 sEVs (sEVs-T/C-Cx43) for 48 h promoted Cx43 gene overexpression in target cells (n=5-7, one-way ANOVA), as well as the expression of the inflammatory genes IL-1β and COX-2 (n=3, Student’s *t* test) (**g**). **h**. Treatment of T/C-28a2 cells with sEVs derived from Cx43-overexpressing T/C-28a2 chondrocytes (sEVs-T/C-Cx43) for 48 h increased both protein and gene expression levels in target chondrocytes. n=4, one-way ANOVA. **i**. When OA-derived chondrocytes were treated with sEVs-T/C-Cx43 for 48 h, EMT-markers gene (n=5-7) and protein (n=3) expression was upregulated, together with decreased gene expression levels of Col2A1 and ACAN (**j**, n=6); one-way ANOVA. Data is expressed as mean ± S.E.M., **P*<0.05, ***P*<0.01, ****P*<0.0001.

**Figure 4. Exosomal Cx43 promotes a senescence inflammatory phenotype in target chondrocytes. a**. Treatment of OA-derived chondrocytes with sEVs derived from Cx43-overexpressing T/C-28a2 chondrocytes (sEVs-T/C-Cx43) for 48 h increased the number of senescence-associated β-galactosidase (SA-β-Gal) positive cells. n=6-9, one-way ANOVA. **b**. The gene expression levels of the senescence marker p16 gene (n=4, Student’s *t* test), as well as the protein levels as p53 (n=3, one-way ANOVA), p21 (n=2) and p16 (n=3, one-way ANOVA) markers were upregulated in OA-derived chondrocytes after a 48-h treatment with sEVs derived from Oligo-treated OA-derived chondrocytes (hCx43-sEVs). **c**. The treatment of OA-derived chondrocytes with sEVs derived from Cx43-overexpressing T/C-28a2 chondrocytes (sEVs-T/C-Cx43) for 48 h lead to increased p53 protein levels (n=3, one-way ANOVA) and the gene expression of p16 (n=5, one-way ANOVA) and p21 (n=4, one-way ANOVA). **d**. Schematic representation and timeline followed for siRNA transfection in Cx43-overexpressing T/C-28a2 chondrocytes. siRNA targeting p53 (sip53) and a siRNA control (siSCR) were transfected into the Cx43-overexpressing T/C-28a2 cell line. sEVs were collected 72 h after transfection and immediately used to treat primary chondrocytes for 48h. **e**. P53 RNA-analysis in sip53 knockdown in T/C28a2 chondrocytes overexpressing Cx43, compared to siSCR in the same cell type. n=3, Student’s *t* test. **f.** RNA expression levels of p53, p21 in OA primary chondrocytes after treatment with sEVs for 48h. **g**. Heat map showing the expression levels of different p53-targets and NF-kB SASP factors after sEVs treatment for 48h, n=3. **h**. sEVs derived from Cx43-overexpressing T/C-28a2 chondrocytes (sEVs-T/C-Cx43) promoted NF-kβ activation in OA-derived chondrocytes, as detected by the increased number of nuclear NF-kβ positive cells. n=3, one-way ANOVA. **i**. RNA expression analysis of different SASP-components showed upregulation in OA-derived chondrocytes after 48-h treatment with sEVs-T/C-Cx43. n=4-7, one-way ANOVA. Data is expressed as mean ± S.E.M., **P*<0.05, ***P*<0.01, ****P*<0.0001.

**Figure 5.** **Proteomic analysis of proteins isolated from sEVs derived from T/C-28a2 chondrocytes with (sEVs-T/C-Cx43) or without (sEVs-T/C) Cx43**. **a.** sEVs were isolated by ultracentrifugation and the protein content was analyzed by mass spectrometry (TripleTOF 6600 System). Venn diagram showing the number of proteins quantified in both groups (sEVs-T/C in the left, sEVs-T/C-Cx43 in the right), and the heatmap of upregulated and downregulated proteins in the bottom. **b.** Volcano plot (VolcaNoseR) illustrating the spectral count fold change and its significance in the common proteins shared by sEVs-T/C and sEVs-T/C-Cx43. Overrepresented proteins in sEVs-T/C-Cx43 are displayed as red dots, while blue dots represent proteins that are increased in the sEVs-T/C group. **c.** Heatmaps representing the spectral count values and the -10log p-value of proteins involved in NF-kß transcription factor regulation, mitochondrial membrane permeabilization and organization, ROS response, immune response, apoptosis, mesenchymal cell differentiation and senescence. Black lines correspond to -10log p-value of sEVs-T/C and red lines to -10log p-value sEVs-T/C-Cx43.

**Figure 6.**   **Proteomic analysis of proteins isolated from sEVs derived from untreated human osteoarthritic chondrocytes (sEVs) or Cx43-enriched after oligomycin treatment (hCx43-sEVs)**. **a.** Venn diagram indicating the number of proteins identified in the proteomic analysis, and the heatmap of enriched pathways present in each group (n = 4). 114 proteins were exclusively found in sEVs isolated from OA-derived chondrocytes (left) and 161 were only identified in hCx43-sEVs (right). 499 common proteins (middle circle) were identified in both samples. Heatmaps represents significant function and pathways enriched in exclusive and common detected proteins, and analyzed using STRING enrichment analysis (Gene Ontology and KEGG) based on quantitatively upregulated and downregulated proteins. **b.** Volcano plot (VolcaNoseR) illustrating the correlation of fold change derived from the semiquantitative parameter spectral count and its significance, of the common proteins presents in both groups (sEVs from OA-derived chondrocytes -derived and sEVs derived from hCx43).

**Figure 7. sEVs secreted by OA-derived chondrocytes promote inflammation and senescence in bone cells. a**. Red Dil-labelled sEVs isolated from OA-derived chondrocytes were detected in bone cells (BC) after 48-h treatment. Cx43 protein (middle; n=3, Student’s *t* test) and RNA (right, n=4; Student’s *t* test) levels were upregulated in bone cells treated with sEVs derived from OA-derived chondrocytes, as determined by western blot and RT-qPCR, respectively. **b**. RNA levels of the EMT-transcription factor Twist-1 was also significantly upregulated in bone cells after a 48-h treatment with sEVs from OA chondrocytes. n=4, Student’s *t* test. **c**. Senescence-associated β-galactosidase activity (SA-β-Gal) was detected by microscopy (left; n=4, Student’s *t* test) and flow cytometry (right; n=4 Student’s *t* test) in bone cells treated for 48 h with sEVs derived from OA-derived chondrocytes. **d**. Western blot analysis showed increase p53 protein levels in bone cells treated with sEVs isolated from OA-derived chondrocytes. On the right, gene expression levels of the senescence markers p16 and p21 were upregulated in BC after a 48-h treatment with sEVs from OA chondrocytes. n=4, Student’s *t* test. **e**. Treatment of BC with sEVs isolated from OA-derived chondrocytes for 48 h promoted NF-kβ nuclear translocation, as detected by immunofluorescence. n=3, Student’s *t* test. **f**. Overexpression of SASP factors IL-1β, IL-6, COX-2 and MMP-3 in BC treated for 48 h with sEVs isolated from OA-derived chondrocytes. n=4, Student’s *t* test. Data is expressed as mean ± S.E.M., **P*<0.05, ***P*<0.01, ****P*<0.0001.

**Figure 8. sEVs derived by OA-derived chondrocytes promote inflammation and senescence in synovial cells. a**. Red Dil-labelled sEVs isolated from OA-derived chondrocytes were detected in synovial cells (SV) after 48-h treatment. Cx43 protein and RNA levels were upregulated in synovial cells (SV) treated with sEVs derived from OA-derived chondrocytes, as detected by western blot and RT-qPCR, respectively. n=4; Student’s *t* test. **b**. RNA levels of the EMT-transcription factor Twist-1 was significantly upregulated in synovial cells after a 48-h treatment with sEVs isolated from OA-derived chondrocytes. n=4, Student’s *t* test. **c**. Senescence-associated β-galactosidase activity (SA-β-Gal) levels were upregulated synovial cells treated for 48 h with sEVs derived from OA-derived chondrocytes. n=3, Student’s *t* test. **d**. The gene expression levels of the senescence markers p16 and p21 were upregulated in SV after a 48-h treatment with sEVs derived from OA-derived chondrocytes. n=4, Student’s *t* test. **e**. Treatment of synovial cells (SV) with sEVs isolated from OA-derived chondrocytes promoted NF-kβ nuclear translocation, as detected by immunofluorescence. n=3, Student’s *t* test. **f**. Overexpression of SASP factors IL-1β, IL-6, COX-2 and MMP-3 in SV treated for 48 h with sEVs isolated from OA-derived chondrocytes. n=4-5, Student’s *t* test. Data is expressed as mean ± S.E.M., **P*<0.05, ***P*<0.01, ****P*<0.0001.

**Figure 9. sEVs derived from Cx43-overexpressing human chondrocytes promote OA-phenotype in bone and synovial cells. a**. Senescence-associated β-galactosidase activity (SA-β-Gal) levels were upregulated in both BC (left) and SV (right) treated for 48 h with sEVs derived from Cx43-overexpressing T/C-28a2 chondrocytes. n=3, one-way ANOVA. **b**. Treatment of BC (left, n=4) and SV (right, n=4-5) with sEVs derived from Cx43-overexpressing T/C-28a2 chondrocytes (sEVs-T/C-Cx43) for 48 h increased p16 and p21 gene expression levels. One-way ANOVA. **c**. IL-1β, IL-6 and MMP-3 expression was upregulated in both bone cells (left) and synovial f cells (right) after treatment with sEVs derived from Cx43-overexpressing T/C-28a2 chondrocytes (sEVs-T/C-Cx43) for 48 h. n=4, one-way ANOVA. **d**. Treatment of BC (n=4) with sEVs derived from Cx43-overexpressing T/C-28a2 chondrocytes (sEVs-T/C-Cx43) for 48 h increased SNAI1 and SNAI1 gene expression levels. One-way ANOVA. **e**. The treatment of BC (left) and SV (right) with sEVs isolated from Cx43-overexpressing T/C-28a2 chondrocytes (sEVs-T/C-Cx43) promoted NF-kβ nuclear translocation. n=4-5, one-way ANOVA. Data is expressed as mean ± S.E.M., **P*<0.05, ***P*<0.01, ****P*<0.0001.

**Supplementary Figure 1.** Immunofluorescence for Twist-1 (red) showed a significant nuclear localization of this EMT-related protein in OA-derived chondrocytes treated with hCx43-sEVs for 48 h (n=6, one-way ANOVA). Nuclei were stained with DAPI (blue). Data is expressed as mean ± S.E.M., ***P*<0.01.

**Supplementary Figure 2.** Immunofluorescence for Twist-1 (red) showed a significant nuclear localization of this EMT-related protein in synovial cells (SV) treated with exosomes isolated from Cx43-overexpressing T/C-28a2 chondrocytes (sEVs-T/C-Cx43) for 48 h (n=6, one-way ANOVA). Nuclei were stained with DAPI (blue). Data is expressed as mean ± S.E.M., ****P*<0.0001.

**Supplementary Figure 3.** Uncropped western-blot images related to the images depicted in the main Figures.

**Supplementary tables:**

**Table S1.** List of primer sequences (5’-3’) used in this study for qRT-PCR experiments.

**Table S2.** List of the total amount of proteins identified in sEVs-T/C and sEVs-T/C-Cx43. The table includes the gene name, the protein name, the false discovery rate (FDR), sequence coverage at 95% (%Cov(95)) and peptide number (PN). Only proteins whit more than 1 PN and and <1% FDR in sEVs-T/C and >0,85 FDR in sEVs-T/C-Cx43 were selected for this study. Empty boxes indicate that the protein has only been identified in one group and therefore was excluded.

**Table S3**. List of proteins identified in sEVs-T/C and sEVs-T/C-Cx43 using LC-MS/MS. The table includes the gene name, protein accession name, a description of the protein, spectral counts of each protein per group, spectral counts related p-values and fold change between both groups, calculated as sEVs-T/C-Cx43 vs. sEVs-T/C ratio. Proteins are listed from the highest to the lowest spectral count fold change.

**Table S4**. List of proteins identified exclusively in sEVs-T/C-Cx43 using LC-MS/MS. The table includes the gene name, protein accession name, a description of the protein, spectral counts of each protein per group and spectral counts related p-values. Proteins are listed from the highest to the lowest detected spectral counts.

**Table S5**. List of proteins identified exclusively in sEVs-T/C using LC-MS/MS. The table includes the gene name, protein accession name, a description of the protein, spectral counts of each protein per group and spectral counts related p-values. Proteins are listed from the highest to the lowest detected spectral counts.

**Table S6.** Example of differentially expressed proteins in sEVs-T/C and sEVs-T/C-Cx43. The table shows the spectral counts, p-values (-10logp) and fold change of each protein (calculated as sEVs-T/C-Cx43 vs. sEVs-T/C ratio). Only proteins with >95% probability were selected for this study.

**Table S7.** List of the total amount of proteins identified in sEVs and hCx43-sEVs derived from OA patients using LC-MS/MS. The table includes the gene name, the protein name, the false discovery rate (FDR), sequence coverage at 95% (%Cov(95)) and peptide number (PN). Only proteins whit more than 1 PN and >0,72 for hCx43-sEVs and <1% FDR for sEVs were selected for this study. Empty boxes indicate that the protein has only been identified in one group and therefore was excluded.

**Table S8**. List of proteins identified in sEVs isolated from OA patients with basal (sEVs) or high levels of Cx43 (hCx43-sEVs). The table includes the gene name, protein accession name, a description of the protein, spectral counts of each protein per group, spectral counts related p-values and fold change between both groups. Proteins are listed from the highest to lowest spectral count fold change.

**Table S9**. List of proteins identified exclusively in hCx43-sEVs using LC-MS/MS. The table includes the gene name, protein accession name, a description of the protein, spectral counts of each protein per group and spectral counts related p-values. Proteins are listed from the highest to the lowest expression detected spectral counts.

**Table S10**. List of proteins identified exclusively in OA chondrocytes-derived sEVs using LC-MS/MS. The table includes the gene name, protein accession name, a description of the protein, spectral counts of each protein per group and spectral counts related p-values. Proteins are listed from the highest to the lowest expression detected spectral counts

**Table S11.** Example of differentially expressed proteins in derived sEVs from OA chondrocytes with high (hCx43-sEVs) and low Cx43 (sEVs) levels. The table shows the spectral counts, p-values (-10logp) and fold change (calculated as sEVs vs. hCx43-sEVs) of each protein. Only proteins with >95% probability were selected for this study.
