# Supplementary material for: Extracellular vesicles enriched in connexin 43 promote a senescent phenotype in bone and synovial cells contributing to osteoarthritis progression
Source: Cell Death Dis. 2022 Aug 5;13(8):681. doi: 10.1038/s41419-022-05089-w (PMC9355945; doi:10.1038/s41419-022-05089-w)
Supplement: Supplementary file 4 — Supplementary Legends [file 41419_2022_5089_MOESM4_ESM.docx]

**Supplementary figures**

**Supplementary Figure 1.** Immunofluorescence for Twist-1 (red) showed a significant nuclear localization of this EMT-related protein in OA-derived chondrocytes treated with hCx43-sEVs for 48 h (n=6, one-way ANOVA). Nuclei were stained with DAPI (blue). Data is expressed as mean ± S.E.M., ***P*<0.01.

**Supplementary Figure 2.** Immunofluorescence for Twist-1 (red) showed a significant nuclear localization of this EMT-related protein in synovial cells (SV) treated with exosomes isolated from Cx43-overexpressing T/C-28a2 chondrocytes (sEVs-T/C-Cx43) for 48 h (n=6, one-way ANOVA). Nuclei were stained with DAPI (blue). Data is expressed as mean ± S.E.M., ****P*<0.0001.

**Supplementary Figure 3.** Uncropped western-blot images related to the images depicted in the main Figures.

**Supplementary tables**

**Table S1.** List of primer sequences (5’-3’) used in this study for qRT-PCR experiments.

**Table S2.** List of the total amount of proteins identified in sEVs-T/C and sEVs-T/C-Cx43. The table includes the gene name, the protein name, the false discovery rate (FDR), sequence coverage at 95% (%Cov(95)) and peptide number (PN). Only proteins whit more than 1 PN and and <1% FDR in sEVs-T/C and >0,85 FDR in sEVs-T/C-Cx43 were selected for this study. Empty boxes indicate that the protein has only been identified in one group and therefore was excluded.

**Table S3**. List of proteins identified in sEVs-T/C and sEVs-T/C-Cx43 using LC-MS/MS. The table includes the gene name, protein accession name, a description of the protein, spectral counts of each protein per group, spectral counts related p-values and fold change between both groups, calculated as sEVs-T/C-Cx43 vs. sEVs-T/C ratio. Proteins are listed from the highest to the lowest spectral count fold change.

**Table S4**. List of proteins identified exclusively in sEVs-T/C-Cx43 using LC-MS/MS. The table includes the gene name, protein accession name, a description of the protein, spectral counts of each protein per group and spectral counts related p-values. Proteins are listed from the highest to the lowest detected spectral counts.

**Table S5**. List of proteins identified exclusively in sEVs-T/C using LC-MS/MS. The table includes the gene name, protein accession name, a description of the protein, spectral counts of each protein per group and spectral counts related p-values. Proteins are listed from the highest to the lowest detected spectral counts.

**Table S6.** Example of differentially expressed proteins in sEVs-T/C and sEVs-T/C-Cx43. The table shows the spectral counts, p-values (-10logp) and fold change of each protein (calculated as sEVs-T/C-Cx43 vs. sEVs-T/C ratio). Only proteins with >95% probability were selected for this study.

**Table S7.** List of the total amount of proteins identified in sEVs and hCx43-sEVs derived from OA patients using LC-MS/MS. The table includes the gene name, the protein name, the false discovery rate (FDR), sequence coverage at 95% (%Cov(95)) and peptide number (PN). Only proteins whit more than 1 PN and >0,72 for hCx43-sEVs and <1% FDR for sEVs were selected for this study. Empty boxes indicate that the protein has only been identified in one group and therefore was excluded.

**Table S8**. List of proteins identified in sEVs isolated from OA patients with basal (sEVs) or high levels of Cx43 (hCx43-sEVs). The table includes the gene name, protein accession name, a description of the protein, spectral counts of each protein per group, spectral counts related p-values and fold change between both groups. Proteins are listed from the highest to lowest spectral count fold change.

**Table S9**. List of proteins identified exclusively in hCx43-sEVs using LC-MS/MS. The table includes the gene name, protein accession name, a description of the protein, spectral counts of each protein per group and spectral counts related p-values. Proteins are listed from the highest to the lowest expression detected spectral counts.

**Table S10**. List of proteins identified exclusively in OA chondrocytes-derived sEVs using LC-MS/MS. The table includes the gene name, protein accession name, a description of the protein, spectral counts of each protein per group and spectral counts related p-values. Proteins are listed from the highest to the lowest expression detected spectral counts

**Table S11.** Example of differentially expressed proteins in derived sEVs from OA chondrocytes with high (hCx43-sEVs) and low Cx43 (sEVs) levels. The table shows the spectral counts, p-values (-10logp) and fold change (calculated as sEVs vs. hCx43-sEVs) of each protein. Only proteins with >95% probability were selected for this study.
