# Supplementary figures and images for: Extracellular vesicles enriched in connexin 43 promote a senescent phenotype in bone and synovial cells contributing to osteoarthritis progression
Source: Cell Death Dis. 2022 Aug 5;13(8):681. doi: 10.1038/s41419-022-05089-w (PMC9355945; doi:10.1038/s41419-022-05089-w)

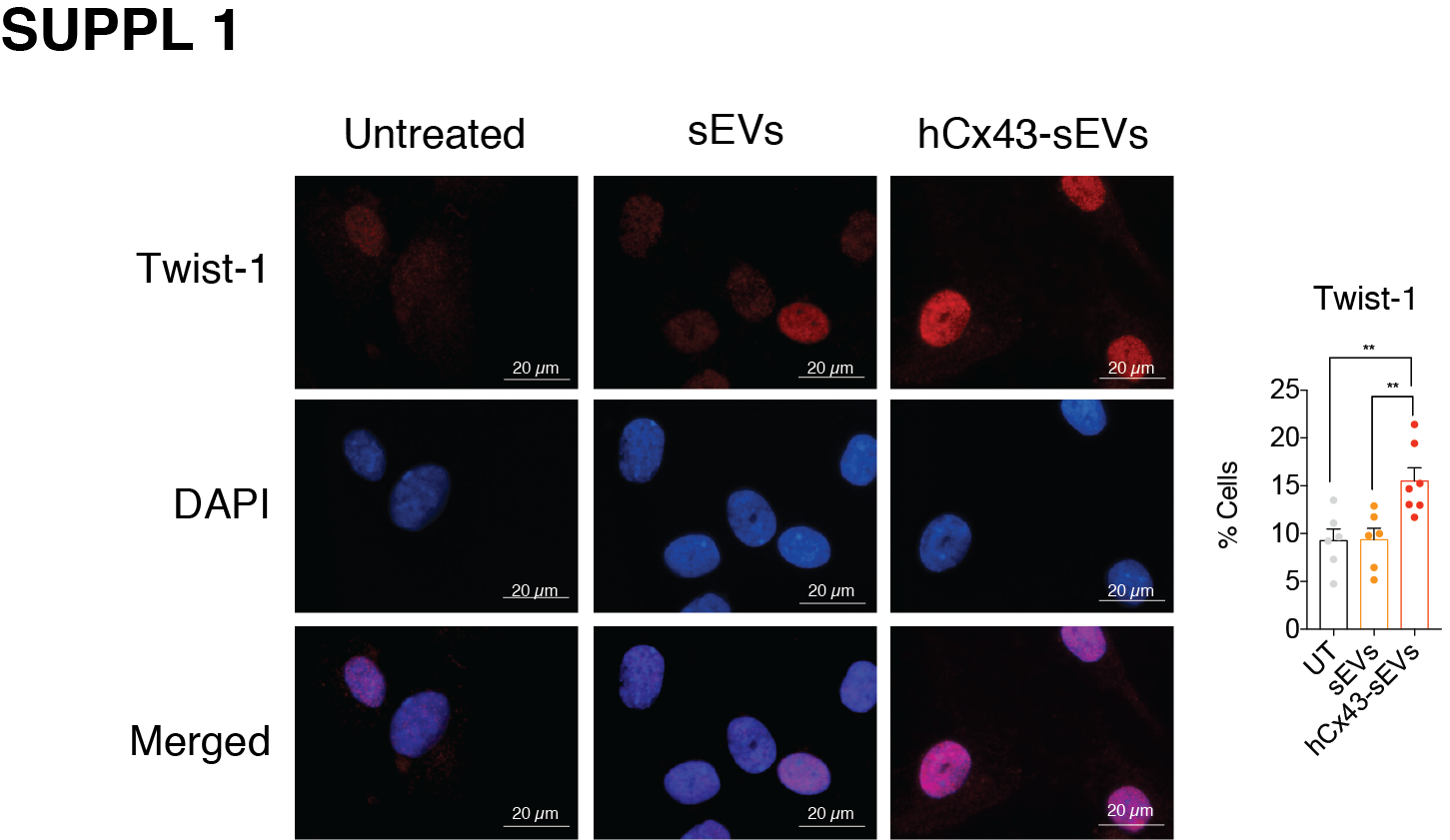

Supplement: Supplementary file 5 — Figure S1 [file 41419_2022_5089_MOESM5_ESM.tif]

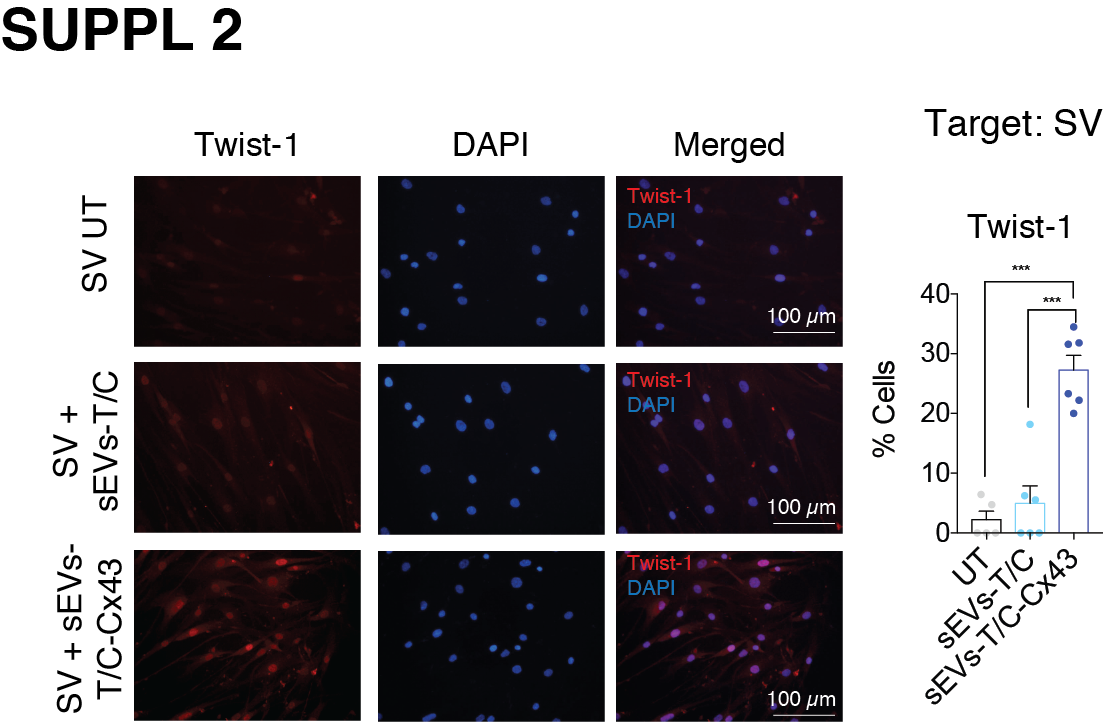

Supplement: Supplementary file 6 — Figure S2 [file 41419_2022_5089_MOESM6_ESM.tif]

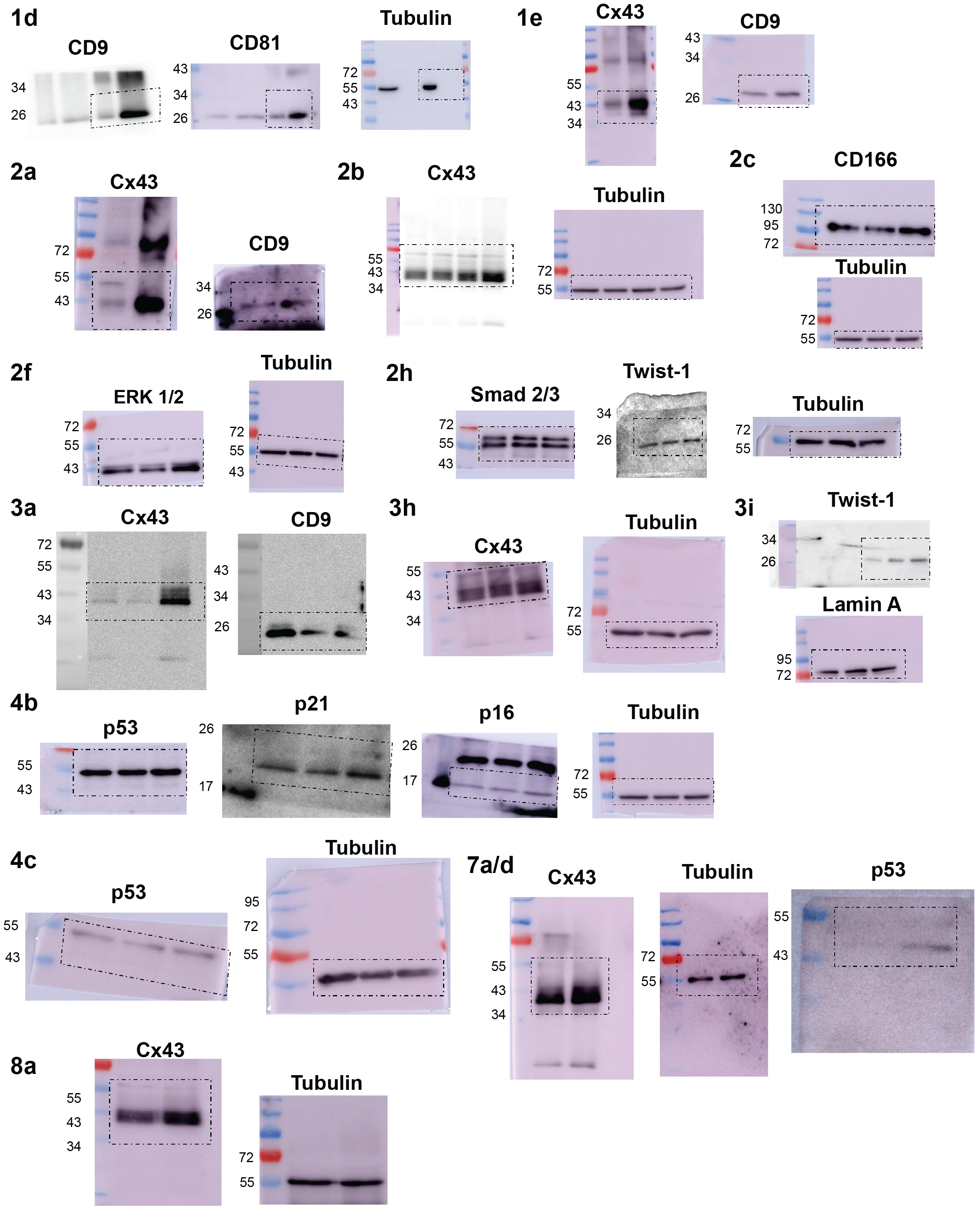

Supplement: Supplementary file 7 — Figure S3 [file 41419_2022_5089_MOESM7_ESM.tif]
